# Materials Engineering to Help Pest Control: A Narrative Overview of Biopolymer-Based Entomopathogenic Fungi Formulations

**DOI:** 10.3390/jof9090918

**Published:** 2023-09-12

**Authors:** Marco Friuli, Rebecca Pellegrino, Leonardo Lamanna, Paola Nitti, Marta Madaghiele, Christian Demitri

**Affiliations:** Department of Engineering for Innovation, University of Salento, 73100 Lecce, Italy; rebecca.pellegrino@unisalento.it (R.P.); leonardo.lamanna@unisalento.it (L.L.); paola.nitti@unisalento.it (P.N.); marta.madaghiele@unisalento.it (M.M.); christian.demitri@unisalento.it (C.D.)

**Keywords:** biopolymers, bioinsecticides, entomopathogenic fungi, integrated pest management

## Abstract

Biopolymer-based formulations show great promise in enhancing the effectiveness of entomopathogenic fungi as bioinsecticides. Chitosan and starch, among other biopolymers, have been utilized to improve spore delivery, persistence, and adherence to target insects. These formulations offer advantages such as target specificity, eco-friendliness, and sustainability. However, challenges related to production costs, stability, and shelf life need to be addressed. Recently, biomimetic lure and kill approaches based on biopolymers offer cost-effective solutions by leveraging natural attractants. Further research is needed to optimize these formulations and overcome challenges. Biopolymer-based formulations have the potential to revolutionize pest control practices, providing environmentally friendly and sustainable solutions for agriculture.

## 1. Introduction

The use of synthetic pesticides has contributed significantly to the increase in food production and control of pest populations. However, the excessive and indiscriminate use of these chemicals has led to a range of negative impacts on human health and the environment, including the development of pesticide-resistant pests, the contamination of water sources, and the killing of non-target organisms [1,2]. Therefore, in recent years, there has been growing interest in developing safer and more environmentally friendly alternatives to synthetic pesticides, such as biopesticides [1].

Biopesticides are derived from natural materials and organisms that can control pests and diseases [3]. Among them, entomopathogenic fungi (EPF), naturally occurring soil microorganisms, have gained popularity as a promising alternative to synthetic pesticides due to their specificity toward target insects (they act mainly by contact with the insect), low toxicity to non-target organisms and environmental and health safety [4]. EPF kill insects by infecting and colonizing them to death, and then, they proliferate by using the death insect as substrate growth. This makes EPF very effective bioinsecticides [5,6,7]. Finally, they can prevent the evolution of resistant pest populations, as entomopathogenic fungi have a complex mode of action that makes it difficult for pests to develop resistance [8].

However, the use of entomopathogenic fungi as bioinsecticides is limited by their scarce tolerance to unsuitable field conditions (e.g., UV exposure, high temperature, drought, surface of application etc.), which reduces their field efficiency and persistence obliging to increase the application frequency with an increase in costs [9,10]. To overcome these limitations, researchers have been exploring the use of biopolymer-based formulations [11,12]. Biopolymers are naturally occurring polymers that can be derived from renewable sources such as plants, animals, and microorganisms. These materials are eco-friendly and biodegradable, and they can be used in pest management strategies to enhance the stability, adherence, and persistence of entomopathogenic fungi in field conditions by shielding the EPF from environmental stressors and providing a suitable microenvironment for their growth, survival and action against target insects [13]. Biopolymer-based formulations are also eco-friendly and biodegradable, making them a sustainable alternative to synthetic insecticides that can persist in the environment for long periods, causing harm to non-target organisms [14]. They can be developed from renewable sources, such as plant and animal materials, thus reducing reliance on non-renewable resources [15].

Consequently, biopolymer-based formulations are studied as a possible solution to improve EPF field efficacy by reducing the initial amount of bioinsecticide required (e.g., the initial conidial concentration) and frequency of application, making EPF more cost-effective. Furthermore, the effect of the biopolymer’s protection could play a role in extending the EPF-based products’ shelf life, which is another limiting factor in the diffusion of EPF as a biopesticide [16].

In this review, we will present the main studied (eventually market available) biopolymer-based formulations of entomopathogenic fungi. We will focus on their preparation, advantages, and drawbacks. The review will not focus on the efficacy of the formulation since very few of them have been tested in the field or studied in comparison to standard formulations. This review aims to highlight the possibility of coupling EPF and biomaterials to improve the general performances of biopesticides, pointing to having cost-effective products that are able to compete against chemical pesticides on the market.

## 2. Method

The search method for this literature review involved a systematic search on electronic databases, including PubMed, Web of Science, and Scopus, using a combination of relevant keywords and search terms such as “biopolymer-based formulations”, “entomopathogenic fungi”, “bioinsecticides”, “cost-effectiveness”, “environmental impact”, “sustainability”, “renewable resources”, and “agriculture”. The search was limited to articles published in English between 2015 and 2022, and only peer-reviewed journal articles were considered. Both primary research articles and review articles were included.

The search was conducted in three stages. In the first stage, an initial search was performed using the keywords and search terms listed above, and the results were screened by title and abstract. In the second stage, the full-text articles were reviewed to determine their relevance to the topic. In the third stage, the reference lists of the selected articles were manually searched to identify additional sources that were not captured in the initial search.

The inclusion criteria for articles were relevance to the topic, scientific rigor, and publication in high-impact factor peer-reviewed journals. The exclusion criteria were articles that were not related to the topic, lacked scientific rigor, published in non-peer-reviewed journals or conference proceedings, or written in languages other than English.

After the three-stage search, a total of 58 articles were selected and used to inform the content of this literature review. These articles included primary research studies, review articles, and meta-analyses that addressed various aspects of biopolymer-based formulations of entomopathogenic fungi, including their effectiveness as bioinsecticides, cost-effectiveness, environmental impact, and potential for sustainable agriculture. The selected articles were critically evaluated and synthesized to provide a comprehensive overview of the topic.

## 3. Biopolymer-Based Formulations of Entomopathogenic Fungi

Biopolymer-based formulations are innovative techniques for the delivery of entomopathogenic fungi as bioinsecticides, making them a promising strategy in integrated pest management (IPM) [17] useful to overcome some of the main drawbacks that still limit the employment of fungi in pest control.

Several biopolymers have been used to develop formulations of entomopathogenic fungi, including cellulose derivatives (e.g., hydroxyethylcellulose, carboxymethylcellulose), chitosan, alginate, and starch [18,19]. Biopolymers can improve the performances of the EPF by extending conidial survival and the active period in field conditions. Furthermore, they can enhance the interaction between the EPF and target insect (e.g., in lure and kill formulations), including the stability, adherence, and persistence of the fungal spores on the insect’s cuticle, thus increasing EPF’s efficacy as bioinsecticides [20].

Encapsulation techniques are an explicative example. Microencapsulation and nanoencapsulation involve the incorporation of the fungi into small polymer-made spheres or capsules that act as protective barriers around the entomopathogenic fungi, shielding them from environmental stressors (such as UV radiation and desiccation). Encapsulation enhances conidial stability and survival in field conditions, fungal adherence and sporulation on the cuticle of the target insect, product shelf life, and consequently the global efficacy of EPF as bioinsecticides [21].

Alginate is the most employed for the encapsulation and production of granules, nano or microspheres containing *Metarhizium anisopliae* and *Beauveria bassiana* conidia [22,23]. However, starch and chitosan have also been used to develop microspheres containing *Beauveria bassiana* spores [24].

Despite the advantages of biopolymer-based formulations, there are still several challenges associated with their development and use. Firstly, the cost of production can be higher compared to standard formulations (e.g., EPF wettable powder) and synthetic polymers-based formulations due to the need for specialized equipment and processes and to the sourcing of raw materials [25]. Secondly, the stability of the formulations can be affected by environmental factors, such as temperature and humidity, which can impact their efficacy [26]. Thirdly, the shelf life of the formulations can be shorter than synthetic polymers due to moisture, oxygen, and microbial contamination that can accelerate their degradation process and impact their availability and accessibility to farmers [27]. Furthermore, the viscosity and solubility of biopolymers can also impact their formulation process, requiring the use of specific additives to achieve desired properties (e.g., plasticizers, gelling agents, organic solvents, etc.) [28]. Additionally, the delivery of biopolymer-based formulations can be more challenging than standard or synthetic polymers-based formulations, as the particle size and surface charge can impact the efficacy of the product [20].

For these reasons, although biopolymer-based formulations of entomopathogenic fungi hold great potential for the development of effective and environmentally friendly bioinsecticides, further research is needed to address the challenges associated with their development and to optimize their use in agricultural systems to have a better balance between the cost and benefit of their employment.

Many strategies can be useful to increase cost efficacy. For example, one approach is to explore alternative sources of biopolymers that may be more cost-effective and readily available, such as waste materials and agricultural residues. Alternatively, recently, the concept of biomimetic lure and kill approaches holds promise for developing cost-effective biopolymer-based formulations [13,29]. They are designed to mimic the chemical signals released by target insects or to replicate some specific/attractive environmental conditions (humidity, surface morphology, pH, texture of a substrate, etc.), thus increasing their attraction to a specific location, where they are then killed using an entomopathogenic fungus or other bioactive agents [13,29].

By incorporating biopolymers into lure and kill systems, several advantages can be realized. Biopolymers can serve as carriers for attractive compounds or pheromones, enhancing their stability and release characteristics, which improves the efficacy of the lure. Biopolymer matrices can also protect bioactive agents, such as entomopathogenic fungi, from environmental stressors, ensuring their viability and activity when the target insects come into contact with the formulation [13]. By using a biopolymer-based lure to attract insects to a specific location, the amount of bioinsecticide needed can be significantly reduced. This can lead to cost savings in the production and application of bioinsecticides.

**Figure 1:**
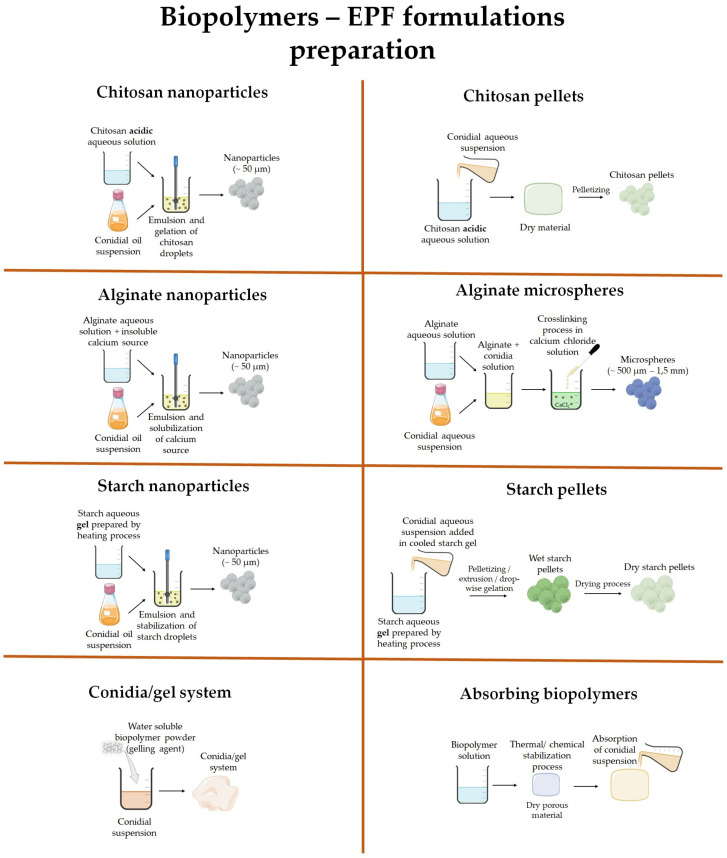
Schematic representation of the main preparation steps for biopolymer-based EPF formulations.

Furthermore, the incorporation of bioactive compounds, such as natural antioxidants and antimicrobial agents, into biopolymer-based formulations can also contribute to their stability and shelf life. These bioactive compounds can help mitigate oxidative damage, inhibit microbial growth, and prevent fungal spore germination during storage. By incorporating these additives, the formulation’s integrity and efficacy can be maintained for longer periods, ensuring its availability and usability by farmers. A collection of studies about this topic is presented in Table 1 and a schematic representation of the preparation has been reported in Figure 1.

### 3.1. Chitosan-Based Formulations

Chitosan-based formulations are an emerging approach in the development of bioinsecticides, in particular for entomopathogenic fungi, as they can enhance their efficacy, persistence and adherence on target insects. Chitosan is a biopolymer that is derived from chitin, which is a naturally occurring polysaccharide that is found in the exoskeletons of crustaceans and insects [38]. Chitosan-based formulations are eco-friendly, biodegradable, and non-toxic, making them an attractive alternative to synthetic pesticides.

One of the most common methods of preparation of chitosan-based formulations is the use of chitosan pellets. Chitosan pellets are small, spherical particles that contain the entomopathogenic fungus and are designed for controlled delivery onto the target insect. Pellets are prepared starting from the dissolving chitosan into an aqueous acetic acid solution containing the EPF. The polymer-conidia solution is dried and then ground into pellets. These pellets have been found to enhance the persistence and adherence of the fungal spores on the insect, resulting in improved mortality rates [39]. The use of chitosan pellets has been shown to be effective in the control of several insect pests, including the Asian citrus psyllid (*Diaphorina citri*) and the diamondback moth (*Plutella xylostella*) [33]. The effectiveness of chitosan pellets can be further enhanced by the addition of adjuvants, such as surfactants, which improve the spreading and wetting of the pellets on the target insect.

Another method of preparation of chitosan-based formulations is the use of chitosan nanoparticles. They are usually prepared by first dissolving chitosan powder into an aqueous acetic acid solution containing the EPF and then pouring this solution dropwise into a dispersion media or adding a stabilizing agent and mixing. Then, the solution is incubated until the reaction is completed and microspheres are recovered by centrifugation.

For example, they have been developed for the delivery of *Metarhizium anisopliae* spores and have been found to be effective in the control of the western flower thrips (*Plutella xylostella*) [34]. Chitosan nanoparticles have also been developed for the delivery of *Trichoderma asperellum* spores and have been found to be effective in the control of three important soil-borne fungal plant pathogens (*Fusarium oxysporum*, *Sclerotium rolfsii* and *Rhizoctonia solani*) [35].

Another technique for the chitosan-based formulation of entomopathogenic fungi is the development of chitosan hydrogels (even though not so common and commercially available). Chitosan hydrogels have been shown to provide a suitable environment for the growth and sporulation of entomopathogenic fungi while also protecting them from environmental stressors such as UV radiation, desiccation, and high temperatures [40]. Usually, a porous dry absorbent chitosan sponge-like material is produced through the thermal stabilization of a previously freeze-dried chitosan solution. Then, the conidial suspension can be inoculated into the material.

Chitosan can also be used as a nutrient or to improve the performances in other biopolymer-based formulations of entomopathogenic fungi. For example, chitosan can be added to alginate beads [41]. The addition of chitosan in alginate beads can enhance their effectiveness, providing a protective barrier around the alginate beads and improving their stability and adhesion to the target insect [42].

Chitosan is a biodegradable and biocompatible polymer, which makes it environmentally friendly and safe for use in agricultural applications [43]. Chitosan is also easily modified to suit specific formulation requirements, such as varying the molecular weight or degree of deacetylation.

However, there are also some drawbacks that need to be considered. Chitosan is a relatively expensive material compared to synthetic polymers, and also, its processing is expensive. This may limit its commercial viability for some applications. Another drawback is the variability in the physical and chemical properties of chitosan, which may be influenced by the pH of the surrounding environment, affecting the performance of the bioinsecticide. Chitosan is positively charged at acidic pH, which may result in reduced efficacy in alkaline environments such as soils with high pH [44]. Furthermore, chitosan needs an acidic environment to be solubilized, which could be aggressive for EPF.

For this reason, the use of chitosan-based formulations may not be suitable for all crops and pests.

In conclusion, chitosan-based formulations have been shown to be an effective and environmentally friendly method for enhancing the efficacy of entomopathogenic fungi as bioinsecticides. Different chitosan-based formulations such as pellets, alginate beads, and hydrogels have been developed for the delivery of entomopathogenic fungi and have shown improved persistence, adherence, and efficacy against target insects. However, the commercial viability of chitosan-based formulations may be limited by its relatively high cost and its sensitivity to pH. Further research is needed to explore the potential of chitosan-based formulations for the development of effective and affordable bioinsecticides.

### 3.2. Alginate-Based Formulations

Alginate-based formulations have gained interest as a promising strategy to improve the effectiveness of entomopathogenic fungi as bioinsecticides. Alginate is a biopolymer extracted from brown seaweed that is composed of linear copolymers of β-D-mannuronic acid and α-L-guluronic acid [45]. Alginate is a biodegradable, biocompatible, edible and environmentally friendly water-soluble material. Alginate became a rigid gel insoluble in water if poured into a solution containing a divalent ions starting a crosslinking reaction (e.g., calcium chloride water solution, called crosslinking agent). This ability is exploited for spherification process (alginate is poured dropwise into the crosslinking solution). It is a technique used to create small, liquid-filled spheres (from nano to microspheres) with a gel-like stable outer shell and a liquid or gel-like core. Its features (such as gelation time, mechanical strength, time of degradation etc.) can also be tuned through the crosslinking reaction. These properties make alginate suitable for a wide range of applications, including pest control [46,47].

Alginate–EPF formulations have low toxicity to non-target organisms, are safe for human health and do not leave harmful residues in the environment [48]. Several alginate-based formulations have been developed for the delivery of entomopathogenic fungi, such as gels and beads, for the control of various insect pests. In general, alginate-based formulations are known to improve the efficacy of entomopathogenic fungi as bioinsecticides by protecting the fungal spores from environmental stressors, enhancing their persistence on the target insect, and prolonging their shelf life.

In a study conducted by Sarma et al., alginate gel formulations containing *Metarhizium anisopliae* spores were found to increase the persistence and viability of the fungal spores over time [49]. Alginate gel formulations were prepared by mixing *Metarhizium anisopliae* spores with sodium alginate water solution, forming a gel. The gel was then extruded into small droplets and allowed to crosslink in a calcium chloride solution, forming spherical beads. The beads were then dried inside a laminar air flow cabinet for 32 to 42 h at 28 ± 2 °C [49].

Another alginate-based formulation was developed for the storage of submerged conidia and microsclerotia (MS) of *Trichoderma asperellum*. The formulation consisted of alginate beads containing spores, which were stored at 8, 25 and 35 °C over 120 days. The formulation was found to be effective in maintaining conidia and MS concentration for freeze-dried beads stored at 8 °C [50].

Another alginate-based formulation containing *Beauveria bassiana* spores was developed for the control of the blood-sucking bug *Triatoma infestans*, which is the main Chagas disease vector in the Southern Cone of Latin America. The formulation consisted of alginate gel beads containing spores, which were tested under semi-field conditions. The fungal microencapsulated formulation caused higher nymph mortality than the unmicroencapsulated fungus and contributed to maintaining the conidial viability throughout the period evaluated under the tested conditions [51]. 

Alginate beads have also been developed for the delivery of *Beauveria bassiana* and *Metarhizium anisopliae* spores against the sugarcane borer, *Diatraea saccharalis*. As previously mentioned, alginate beads were found to protect spores from environmental stressors, such as UV radiation and desiccation, improving their shelf life and efficacy as bioinsecticides [52]. In this case, alginate beads containing both fungi spores were prepared by mixing the spores with a sodium alginate solution and slowly adding calcium chloride solution, forming capsules that were then washed with distilled water and dried at 24 °C for 48 h. The alginate formulation protected the conidia against the radiation until 48 h, because even after exposure, the fungi remained viable. In addition to this, the dry encapsulated conidia *Beauveria bassiana* caused 79.6% mortality of the studied pest, and the *Metarhizium anisopliae* caused only 10% [53].

Several patents have been filed for alginate-based formulations such as bioinsecticides. For example, US Patent 9808768B2 describes an alginate-based formulation containing entomopathogenic fungi for the control of agricultural pests. The formulation is designed to improve the adherence of the fungal spores to the target insect and protect them from environmental stressors. Another patent, US Patent 10425845B2, describes an alginate-based formulation containing *Bacillus thuringiensis* for the control of mosquito larvae. The formulation is designed to release the bacterial spores slowly over time, increasing their persistence and efficacy as a larvicide. A patent application was filed for an alginate-based formulation containing *Beauveria bassiana* spores for the control of the maize weevil (*Sitophilus zeamais*). The formulation was found to be effective in reducing weevil population and increasing grain yield.

There are also commercially available alginate-based formulations for the delivery of entomopathogenic fungi: for example, the product Algibio, which contains alginate beads with *Beauveria bassiana* spores. Algibio has been used for the control of various insect pests, such as the citrus blackfly (*Aleurocanthus woglumi*) and the coffee berry borer (*Hypothenemus hampei*). Algibio has been shown to have high efficacy and a prolonged effect on target insects.

While alginate-based formulations have shown promise as effective delivery systems for entomopathogenic fungi, there are some drawbacks to consider. Alginate-based formulations can be sensitive to pH and temperature changes, which can affect the integrity and stability of the formulation [54]. Additionally, alginate-based formulations may require a high concentration of calcium chloride to maintain the integrity of the beads, which can be costly compared to chemical insecticides, thus limiting their use in certain markets [46]. Furthermore, it is difficult to produce uniform-sized beads or gels, which can affect the consistency and efficacy of the formulation, as smaller beads may not be able to contain enough spores to achieve a high enough concentration for effective control. Another challenge is the limited shelf life of alginate-based formulations, which can be affected by storage conditions and the age of the spores [47].

Overall, alginate-based formulations show promising results as effective and environmentally friendly bioinsecticides. With further development and optimization, they have the potential to become a widely used alternative to conventional insecticides in agriculture.

### 3.3. Starch-Based Formulations

Starch, a biopolymer derived from various plant sources like corn, potato, and rice, has garnered attention in the development of formulations to enhance the efficacy of entomopathogenic fungi as bioinsecticides [55]. These starch-based formulations offer promising strategies to improve the delivery, persistence, and adherence of fungal spores on target insects. This section will provide a detailed exploration of starch-based formulations, including starch pellets and starch microspheres, outlining their advantages, drawbacks, and potential applications.

Starch pellets have been developed as a delivery system for entomopathogenic fungi, specifically *Beauveria bassiana* spores. The use of starch pellets has demonstrated improvements in the persistence and adherence of fungal spores on target insects, leading to enhanced control efficacy and insect mortality rates.

The preparation of starch pellets involves a series of steps to encapsulate entomopathogenic fungal spores within small, spherical particles made of starch. First, a suitable starch source is selected, such as corn, potato, or rice starch. The choice of starch may depend on factors such as the availability, cost, and desired properties of the final formulation. The selected starch is typically mixed with water or a suitable solvent to create a starch gel. The gel is formed by heating the starch–water mixture under controlled conditions, which causes the starch granules to swell and absorb water, resulting in a viscous gel-like consistency. Once the starch gel is formed, it is cooled to a specific temperature range suitable for incorporating the entomopathogenic fungal spores. The spores, either in their suspended form or as a formulated product, are added to the starch gel and thoroughly mixed to ensure uniform distribution. The spore-containing starch gel is then shaped Into small, spherical pellets using various methods, such as extrusion, drop-wise gelation, or pelletization techniques. Extrusion involves forcing the gel through a nozzle or die with the desired pellet size, while drop-wise gelation involves carefully dropping the gel into a cross-linking solution to solidify into spherical pellets. The formed starch pellets are subjected to a drying process to remove excess moisture and for stabilization [37]. Common drying methods include air-drying, freeze-drying, or oven-drying, depending on the specific requirements of the formulation and the preservation of fungal spore viability. Starch pellets promote the persistence and adherence of fungal spores on target insects, allowing for prolonged contact and a steady and sustained supply of fungal spores to the target insects over time, thus increasing the chances of successful infection.

In addition to starch pellets, starch has also been utilized to develop microspheres encapsulating *Beauveria bassiana* spores. These starch-based microspheres provide protection to the fungal spores against environmental stressors such as UV radiation and desiccation, thereby enhancing their shelf life and overall efficacy as bioinsecticides. The microspheres act as a physical barrier, shielding the fungal spores and preserving their viability until they come into contact with the target insects [36].

The preparation of starch microspheres involves the encapsulation of entomopathogenic fungal spores within small, uniformly sized spherical particles composed of starch. First, a starch solution is prepared by dispersing starch in water or an appropriate solvent. The concentration of starch in the solution may vary depending on the desired size and characteristics of the microspheres. Entomopathogenic fungal spores, either in their suspended form or as a formulated product, are prepared separately. The spore suspension, which may contain additives to enhance stability and maintain spore viability during the encapsulation process, is then added to the starch solution, and the mixture is subjected to emulsification or dispersion techniques. These methods aim to disperse the spores uniformly within the starch solution, ensuring that each microsphere contains an adequate number of fungal spores. The spore-containing starch solution is then subjected to droplet formation techniques, such as emulsion-based methods or spray drying. Emulsion-based methods involve mechanically stirring or homogenizing the mixture to form droplets of uniform size. Spray drying involves atomizing the mixture into a spray of fine droplets, which rapidly dries to form solid microspheres, which are typically dried to remove residual moisture. Starch microspheres can be customized and tailored for various entomopathogenic fungi, allowing for a versatile formulation approach to combat different insect pests. They offer improved stability and protection to the encapsulated fungal spores, extending their shelf life and ensuring long-term efficacy. They also safeguard fungal spores from detrimental factors like UV radiation and desiccation, preserving their infectivity and biocontrol potential [56].

It is important to note that specific modifications and variations in the preparation methods of starch-based formulations may exist depending on the desired characteristics, application, and specific research protocols outlined in scientific studies.

For example, crosslinking agents, such as glutaraldehyde or calcium ions, can be incorporated into the starch gel or solution to enhance the stability and mechanical strength of the resulting pellets or microspheres. Crosslinking helps to prevent the disintegration or degradation of the starch-based formulations, improving their shelf life and resistance to environmental conditions [57].

To further enhance the protection and performance of starch-based formulations, additional coatings can be applied to the pellets or microspheres. These coatings may consist of biodegradable polymers, surfactants, or bio-adhesive materials that improve adherence to the target insects, provide controlled release properties, or enhance the compatibility of the formulation with specific crops.

Advanced encapsulation technologies, such as spray drying, fluidized bed coating, or electrostatic encapsulation, have been explored to optimize the preparation of starch-based formulations. These techniques offer precise control over the particle size, morphology, and encapsulation efficiency, resulting in formulations with improved uniformity and performance.

Various additives and adjuvants can be incorporated into the starch-based formulations to enhance their efficacy and functionality. These additives may include surfactants, stabilizers, synergistic agents, or other bioactive compounds that enhance the biocontrol properties of the fungal spores or improve the overall performance of the formulation [58].

The preparation process for starch-based formulations can be further optimized by adjusting various processing parameters, such as the temperature, pH, stirring speed, drying conditions, or concentration of starch and spores. These parameters influence the size, morphology, release kinetics, and stability of the resulting pellets or microspheres.

However, similar to starch pellets, the physical and chemical properties of starch-based microspheres may vary, potentially influencing their performance, consistency and applicability in different agricultural systems. Another drawback is the need of appropriate application techniques and equipment to ensure optimal coverage and efficacy.

## 4. Conclusions and Future Perspectives

In conclusion, the use of entomopathogenic fungi as bioinsecticides is a promising strategy for the control of harmful insects in agriculture.

This literature review highlights some already studied biopolymer-based formulations that could enhance the efficacy of entomopathogenic fungi as bioinsecticides. These formulations, including pellets, granules, and gels, offer several advantages over traditional chemical insecticides, including target specificity, eco-friendliness, and sustainability. Additionally, biopolymer-based formulations are eco-friendly and biodegradable, making them a sustainable alternative to chemical insecticides. However, challenges such as production costs, stability, and shelf life need to be addressed to ensure the practicality and cost-effectiveness of these formulations. These goals could be reached following different approaches. One approach involves seeking alternative sources for biopolymers, particularly those based on cellulose and its derivatives. This could include exploring industrial or agricultural waste streams as potential sources. Utilizing such waste materials not only aligns with sustainability goals but can also lead to cost savings in the production of biopolymer-based formulations. On the other hand, it is equally essential to enhance the performance of these formulations in terms of field durability and effectiveness. For instance, strategies like biomimetic lure and kill, as mentioned in the text, are promising avenues. These approaches aim to increase the attractiveness of the formulations, ultimately improving their duration and efficacy in pest management. By pursuing these dual objectives of sourcing biopolymers more sustainably and enhancing formulation performance, we aim to make significant strides in advancing the field of biopolymer-based pest control solutions.

Another important issue is the scalability of the production of EPF–biopolymers formulations. There are both possibilities and obstacles associated with scalability in this context. Some obstacles could be due to the complexity of the processing of natural materials (e.g., variability in raw materials, processing techniques, and quality control can pose challenges), costs, limitations from regulatory compliance (meeting regulatory requirements and safety standards can be a time-consuming and costly process) and for technical reasons (maintaining product consistency and quality can be technically challenging). Additionally, related to formulation quality, there is the contamination and end-user confidence topic. Contamination by other microbes in EPF (entomopathogenic fungi) biopolymer formulations is indeed an important concern, as it can affect the product’s efficacy and end-user confidence. Biopolymer-based formulations provide a favorable environment for microbial growth due to their composition, which is often rich in organic matter and moisture. This makes them susceptible to contamination by various microorganisms, including bacteria and molds. Microbial contamination can potentially reduce the effectiveness of EPF formulations. Contaminating microorganisms may compete with EPF for resources or produce metabolites that inhibit EPF growth or virulence. In such cases, introducing substances capable of reducing contamination (for example, antimicrobial peptides, chitosan, essential oils, natural resins, metal nanoparticles, etc.) together with stringent quality control measures (e.g., sterile production environment and high-quality raw materials) and stability tests to assess the resistance of EPF formulations to microbial contamination during storage could mitigate this risk. At the same time, the education of end-users about the proper storage and handling of EPF–biopolymer formulations can also help reduce the risk of contamination.

To overcome these limitations, it is crucial to find new polymers, especially those derived from renewable sources like cellulose or agricultural waste, that offer a sustainable alternative to synthetic polymers. This aligns with the growing demand for eco-friendly pest control solutions and could be useful in cost reduction. Furthermore, as biopolymers become more widely available and their production processes improve, economies of scale may lead to cost reductions, making biopolymer-based EPF formulations more economically viable.

Customization and biocompatibility are two significant factors that work in favor of utilizing biopolymers in EPF formulations. In fact, biopolymers can be tailored to specific needs. Researchers can modify their properties to enhance formulation performance, and, at the same time, often exhibit good compatibility with biological agents like EPF. Customization and biocompatibility can enhance the shelf life and effectiveness of the formulations, which may lead to more effective pest control solutions.

Recently, biomimetic lure and kill approaches based on biopolymers have provided a promising avenue for developing cost-effective bioinsecticides by leveraging natural cues and attractants. Further research and development efforts are necessary to overcome these challenges and fully realize the potential of biopolymer-based formulations in integrated pest management strategies. With ongoing advancements, biopolymer-based formulations have the potential to revolutionize pest control practices, offering environmentally friendly and sustainable solutions for agricultural systems.

## Figures and Tables

**Table 1:** Most common biopolymer-based formulations for entomopathogenic fungi.

Bio- Polymer	Entomopathogenic Fungus	Preparation Method	Target Insects	Delivery Method	Commercial Products	References
Alginate	*Metharizium* spp.	Microspheres	Western corn rootworm, cabbage looper, diamondback moth, greenhouse whitefly, Black cutworm, fall armyworm, corn earworm Colorado potato beetle, fall armyworm, corn earworm	Spraying on plant or soil	NoFly WP, Bioinsecticide MC	[30]
	*Beauveria* sp.	Micro-spheres	Cabbage looper, Diamondback moth, Western corn rootworm Chilli thrips, Greenhouse whitefly, Silverleaf whitefly Cabbage looper, European corn borer, fall armyworm	Spraying on plant or soil	Botanigard, Mycotrol NoFly WP, Bioinsecticide MC	[18,24,31]
Chitosan	*Beauveria* sp.	Pellets	Colorado potato beetle, western flower thrips, diamondback moth	Direct application to plant or insect	N/A	[32]
		Beads	Asian citrus psyllid	Direct application to plant or insect	N/A	[33]
	*Metarhizium anisopliae* *Trichoderma asperellum*	Microspheres/Nanoparticles	*Plutella xylostella* *Fusarium oxysporum*, *Sclerotium rolfsii* and *Rhizoctonia solani*	Direct application to plant or insect	N/A	[34,35]
Starch	*Beauveria* sp.	Nano-particles	Chilli thrips, western flower thrips, western corn rootworm Colorado potato beetle, western corn rootworm, diamondback moth European corn borer, fall armyworm, diamondback moth	Spraying on plant or soil	N/A	[36]
	*Metarhizium* spp.	Nano-particles	Western corn rootworm, diamondback moth, cabbage looper	Spraying on plant or soil	N/A	[37]

## Data Availability

Not applicable.
